# Supplementary material for: Resolving the full spectrum of human genome variation using Linked-Reads
Source: Genome Res. 2019 Apr;29(4):635–45. doi: 10.1101/gr.234443.118 (PMC6442396; doi:10.1101/gr.234443.118)
Supplement: Supplemental Material [file supp_gr.234443.118_Supplemental_longranger-2.2.zip › longranger-2.2.tar.bz2!/lr2.2-public/lib/go/src/code.google.com/vitess/third_party/py/mock-1.0.1.tar.gz!/mock-1.0.1/html/search.html]

Search — Mock 1.0.1 documentation


### Navigation

- index
- Mock 1.0.1 documentation »

# Search

Please activate JavaScript to enable the search
functionality.

From here you can search these documents. Enter your search
words into the box below and click "search". Note that the search
function will automatically search for all of the words. Pages
containing fewer words won't appear in the result list.

### Navigation

- index
- Mock 1.0.1 documentation »

© Copyright 2007-2012, Michael Foord & the mock team.
Last updated on Nov 05, 2012.
Created using Sphinx 1.1.3.
